# P-346. *Candida auris:* Colonization, Risk Factors and Antifungal Susceptibility at a Tertiary Healthcare Setting

**DOI:** 10.1093/ofid/ofae631.548

**Published:** 2025-01-29

**Authors:** Kirtana Jonnalagadda, Ayush Goel, Anuj Ajayababu, Immaculata Xess, Gagandeep Singh, Manish Soneja, Ashutosh Biswas

**Affiliations:** All India Institute of Medical Sciences, New Delhi, India, New Delhi, Delhi, India; All India Institute of Medical Sciences, New Delhi, India, New Delhi, Delhi, India; ALL INDIA INSTITUTE OF MEDICAL SCIENCES NEW DELHI INDIA, NEW DELHI, Delhi, India; All India Institute of Medical Sciences, New Delhi, New Delhi, Delhi, India; All India Institute of Medical Sciences, New Delhi, New Delhi, Delhi, India; All India Institute Of Medical Sciences, Delhi, Delhi, India; All India Institute of Medical Sciences, Bhubhaneshwar, Orissa, India

## Abstract

**Background:**

*Candida auris,* a multidrug-resistant nosocomial pathogen, has emerged globally as a major threat to healthcare settings, and several hospital outbreaks continue to be reported. We attempted to study the risk factors associated with *C. auris* colonization and its antifungal susceptibility at a tertiary healthcare center.

1.Distribution of Candida auris isolates

**Figure:**
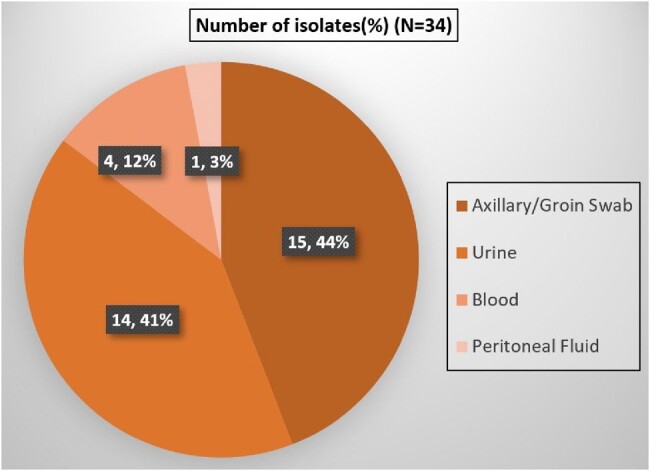

**Methods:**

A prospective observational study was conducted over 18 months. Patients were screened for *Candida auris* colonization by axillary, groin swabs, and urine cultures within 48 hours of admission. Patients with extended hospital stays, exhibiting clinical isolates positive for *C. auris*, were also included. Baseline characteristics and risk factors were assessed. In patients with positive *C. auris* isolates, samples from inanimate surfaces, hands of healthcare workers and patient care attendants were also screened for *C. auris* colonization. Antifungal susceptibility testing was done for all isolates.

**Figure:**
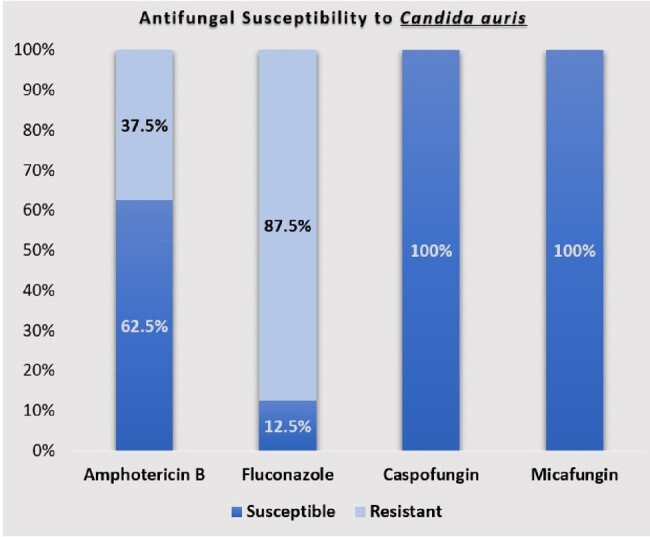

**Results:**

Ninety-nine patients (59% males) were included with a mean age of 43.1±18.06 years. There were 34 *Candida auris* isolates from 20 patients. Sixteen patients (80%) were colonized, and four (20%) had candidemia. Median duration of urinary catheterization (9 vs 3 days [p=0.01]) and prior antifungal exposure(p=0.04) were significant risk factors for *C.auris* colonization. Seventy-eight samples from bed rails, monitors, healthcare workers, and patient-care attendants showed no growth of *C.auris*. Antifungal susceptibility testing showed 87.5% and 37.5% of isolates were resistant to Fluconazole and Amphotericin B respectively, while all isolates were susceptible to Micafungin and Caspofungin.

**Conclusion:**

Prior outbreaks of *Candida auris* at our medical ICU prompted the need for early identification, effective contact precautions, and decolonization to prevent fatal invasive infections. Patients with long-term urinary catheters in situ, prior exposure to antifungals, and prolonged hospital stay were found to be at risk for *C.auris* colonization, warranting further studies to implement routine screening in these high-risk groups.

**Disclosures:**

**All Authors**: No reported disclosures

